# A New *Protocruzia* Species (Ciliophora: Protocruziida) Isolated From the Mariana Trench Area

**DOI:** 10.3389/fmicb.2021.743920

**Published:** 2021-10-20

**Authors:** Chen Liang, Wei Wang, Liang Dong, Irum Mukhtar, Fengping Wang, Jianming Chen

**Affiliations:** ^1^Institute of Oceanography, Minjiang University, Fuzhou, China; ^2^School of Oceanography, Shanghai Jiao Tong University, Shanghai, China

**Keywords:** ciliate, morphology, phylogeny, deep sea, Mariana Trench area

## Abstract

A new species of *Protocruzia*, isolated from the deep-sea Pacific Ocean (>3,000-m depth) in the vicinity of the Mariana Trench, is described based on morphological and molecular data. The systematic status of the ciliate genus *Protocruzia* has long been highly ambiguous, and *Protocruzia* species have been assigned to an independent class until recently. In the present study, we described *Protocruzia marianaensis* sp. n. as a small (25–32 × 14–17 μm *in vivo*) drop-shaped ciliate, with longitudinal furrows along the ciliary rows on the right side, six adoral membranelles, eight somatic kineties, and one macronucleus comprising 7–11 nuclear globules. Phylogenetic analyses inferred from small subunit rRNA gene sequences revealed that seven *Protocruzia* species in the phylogenetic tree formed a fully supported clade representing an independent class. *Protocruzia marianaensis* sp. n. was established to be most closely related to *Protocruzia adhaerens*, with a sequence similarity of 96.64%, and was found to be able to survive at both atmospheric pressure and hydrostatic pressure of 320 bar, thereby indicating effective barotolerance.

## Introduction

Ciliophora are a major, ubiquitous, and diverse group of protozoa. These organisms play an important role in microbial food webs in marine ecosystems (Gentekaki et al., 2014). To date, more than 8,000 nominal species have been reported (Lynn, 2008), including free-living, commensal, and parasitic forms.

The genus *Protocruzia* was established by Faria et al. (1922), and only eight nominal species currently belong to this genus, namely, *Protocruzia adhaerens* (Mansfeld, 1923) Kahl, 1932; *Protocruzia contrax* (Mansfeld, 1923) Kahl, 1932; *Protocruzia depressa* Ammermann, 1968; *Protocruzia granulosa* Kahl, 1933; *Protocruzia labiata* Kahl, 1932; *Protocruzia pigerrima* (Cohn, 1866) Faria et al., 1922; *Protocruzia tuzeti* Villeneuve-Brachon, 1940; and *P. contrax* sensu Song and Wilbert, 1997. Over recent decades, however, the systematic status of the ciliate genus *Protocruzia* has been somewhat ambiguous. Small and Lynn (1985) recognized the family Protocruziidae Jankowski in Small and Lynn, 1985, and established it as the family for the order Protocruziida Jankowski in Small and Lynn, 1985, within the class Karyorelictea (Lynn, 2008), whereas de Puytorac et al. (1987) elevated it to the subclass rank, Protocruziidia, and later placed it within the class Karyorelictea (de Puytorac, 1994). Other studies, however, classified this subclass in the class Spirotrichea rather than Karyorelictea (Small and Lynn, 1985; Adl et al., 2005; Lynn, 2008). Nevertheless, despite several molecular studies on *Protocruzia* having been repeatedly performed, there remains a lack of consensus as to the taxonomic status of this genus (Hammerschmidt et al., 1996; Shin et al., 2000; Li et al., 2010; Gentekaki et al., 2014; Gao et al., 2016; Jiang et al., 2016). Hammerschmidt et al. (1996) and Shin et al. (2000) suggested that the subclass Protocruziidia might be transferred to the class Spirotrichea, whereas Li et al. (2010) rejected their hypothesis, based on the findings of phylogenetic analyses of the small subunit ribosomal RNA gene, internal transcribed spacer 2 sequences, and histone H4 gene. Moreover, Gentekaki et al. (2014) removed the genus *Protocruzia* from Spirotrichea and classified it as incertae sedis in the phylum Ciliophora. In recent years, however, Protocruziea (de Puytorac et al., 1987), comprising the single genus *Protocruzia*, has been recognized as a new class of Ciliophora (Gao et al., 2016).

Estimated to cover approximately 54% of the earth’s surface, the abyssal sea (3,000–6,000 m depth) is the most predominant type of benthic environment (Gage and Tyler, 1991). In recent years, an increasing number of deep-sea investigations have been conducted to study the biodiversity in abyssal areas; however, only a few studies have focused on ciliates. Among those that have been conducted, Hausmann et al. (2002) isolated and cultivated 35 ciliate species from sediments collected in the deep Mediterranean Sea, whereas Schoenle et al. (2017) claimed that ciliate records obtained from the deep sea (depths > 1,000 m) in the Pacific Ocean generally contain representatives from all major groups of Ciliophora. Despite the limited number, these studies did, nevertheless, tend to indicate that a diverse range of ciliate species inhabit the deep sea. However, the data on ciliates from the deep sea remain limited, and most previous studies have been based on molecular analyses, with little in the way of morphological data being presented.

Limited food availability and low temperature would presumably restrict the ciliate colonization of deep-sea habitats. Consequently, low abundances of ciliates and the difficulty in keeping specimens alive during the transfer from regions of high hydrostatic pressure tend to present substantial obstacles to collecting and preserving a sufficient number of samples. Accordingly, it is perhaps not surprising that only a few species have been successfully collected from the deep sea and cultivated in a laboratory setting (Schoenle et al., 2017; Živaljić et al., 2020a).

All previous studies on *Protocruzia* species have focused on populations inhabiting relatively shallow nearshore waters. In the present study, however, we succeeded in isolating and subsequently cultivating a new *Protocruzia* species from a depth of 3,000 m in the vicinity of the Mariana Trench. Moreover, based on morphological and phylogenetic analyses, we were able to morphologically characterize the new *Protocruzia* species and elucidate its phylogenetic position.

## Materials and Methods

### Sampling, Isolation, and Cultivation

**FIGURE 1:**
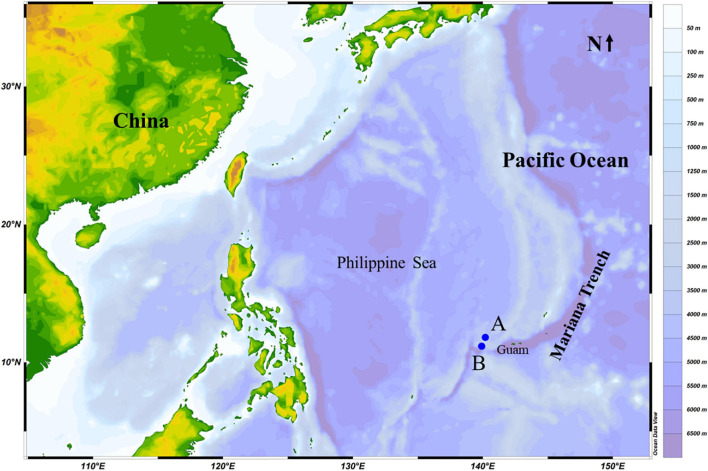
Sampling stations **(A,B)** in the Mariana Trench area (Pacific Ocean) in October 2019.

Samples were collected in an area near the island arc zone in the West Pacific Ocean during a deep-sea expedition onboard a manned submersible vehicle “Shenhai Yongshi” deployed by the research vessel Tansuo Yihao. Samples from stations A (11°13′9″N, 139°55′50″E) and B (11°47′49″N, 140°7′6″E) were collected on the 8th and 23rd of October 2019, respectively (Figure 1). The bottom depths at these two stations were 3,144 and 3,092 m, respectively, and the temperature of these two sampling sites were around 4°C. The map shown in Figure 1 was produced using Data Ocean View (Schlitzer, 2003).

Deep-sea sediments were collected using push corers (outer diameter 7 cm; length 32 cm) operated from the manned submersible vehicle. A closing mechanism at the top and bottom of the cores reduces the risk of contamination with organisms and cysts from upper water layers. The cores, which were collected intact, did not appear to undergo any significant modification during sampling and were stored vertically prior to subsequent subsectioning. We accordingly assume that the likelihood of contamination will have been negligible. Upper waters (34 PSU) immediately above the sediments were also collected at each station. On being returned to the deck of the research vessel, the cores were immediately processed. Core samples extracted from the top 16 cm of sediments were cut into a series of subsections at 2-cm intervals, and a sample from each of these subsections was placed separately into a Petri dish (diameter 60 mm). To the 0–2-cm subsample, we added 20 ml of the corresponding *in situ* collected water samples, whereas samples from the progressively deeper subsections (2–4, 4–6, 6–8, 8–10, 10–12, 12–14, and 14–16 cm) were mixed with 20 ml in-house-prepared sterilized artificial seawater (NaCl, 26.518‰; MgCl_2_, 2.447‰; MgSO_4_, 3.305‰; CaCl_2_, 1.141‰; KCl, 0.725‰; NaHCO_3_, 0.202‰; and NaBr, 0.083‰). To each Petri dish, we also added both one autoclaved grain and shrimp bait to promote the growth of bacteria in water or sediments. In the laboratory, ciliates were detected exclusively in the 0–2-cm subsamples collected from both stations A and B, and single-cell cultivation was conducted using sterilized sea salt (brand “red sea salt”, Red Sea, Israel) water, with the salinity being reduced to 30 PSU for continuous culture at room temperature (25°C).

### Morphological Characterization

Live cells were observed under a Leica microscope with differential interference contrast (Leica Instruments Inc., Wetzlar, Germany). Photographs of the ciliates were obtained using a DFC450 C camera system, and at least 20 individuals were measured (i.e., body length and width) using supporting software (Leica Application Suite X). The protargol staining method (Wilbert, 1975) was used to determine the ciliary pattern and nuclear apparatus. Counts and measurements of the stained specimens were performed at ×1,000 magnification, and drawings were based on photomicrographs and freehand sketches. The systematics and terminology adopted in the present study are mainly based on Lynn (2008) and Gao et al. (2016).

### DNA Extraction, Polymerase Chain Reaction Amplification, and Gene Sequencing

Polymerase chain reactions (PCRs) were performed for genomic sequencing of *Protocruzia marianaensis* sp. n. DNA samples were obtained from three to five cells that were selected from single-cell cultures of station A or B and washed several times with sterilized seawater to ensure no contamination. Total genomic DNA was extracted from these cells using a REDExtract-N-Amp Tissue PCR Kit (Sigma-Aldrich, St. Louis, MO, United States), and using this as a template, we amplified the small subunit (SSU) rDNA with the universal primers Euk A (5′-AACCTGGTTGATCCTGCCAGT-3′) and Euk B (5′-TGATCCTTCTGCAGGTTCACCTAC-3′) (Medlin et al., 1988). The typical amplification profile consisted of pre-denaturation at 94°C for 5 min and then 40 cycles of 95°C for 30 s, 60°C for 1 min, and 72°C for 1 min, followed by a final extension at 72°C for 10 min. Purified PCR products of the appropriate size (∼1,800 bp) were inserted into a pMD19-T vector (Takara Bio Inc., Shiga, Japan) and sequenced using an ABI PRISM 3730 automatic sequencer (Applied Biosystems, Foster City, CA, United States).

### Phylogenetic Analyses

The dataset used for phylogenetic analysis was generated using the sequence data obtained in the present study, along with the sequences from previously characterized organisms available in the GenBank database. Alignments were processed using MAFFT v7.471 (Katoh and Standley, 2013), with a total of 55 aligned sequences being used to construct a phylogenetic tree. Sequences of species in the classes Karyorelictea and Heterotrichea were used as the outgroups. For maximum likelihood (ML) analysis, we used the TIM2 + F + R3 model selected using ModelFinder (Kalyaanamoorthy et al., 2017) and computed using IQ-TREE v1.6.12 (Nguyen et al., 2015) with 1,000 bootstrap replicates. Bayesian inference (BI) analysis was performed using MrBayes v3.2.7 (Ronquist et al., 2012) based on the GTR + I + G model. Markov chain Monte Carlo simulations were run for 4,000,000 generations with an initial burn-in of 25% of the total number.

### Survival Analysis

To determine the survival rates of *P. marianaensis* sp. n. at hydrostatic pressures, we used a hydrostatic pressure system [consisting of an air compressor, stainless steel chamber, and pressure control console (Supplementary Figure 1)] to simulate deep-sea high pressure in the sampling area. The hydrostatic pressure was gradually increased up to 320 bar, which approximates to the pressure recorded at the depths of stations A and B. Prior to the experiment, cultures were adapted for 2 days at a temperature of 4°C in the absence of light. The ciliates were studied during their logarithmic growth phase. Single individual and bacterial culture media were placed in a 200-μl Eppendorf tube (Eppendorf, Wesseling, Germany) with a man-made tiny hole on the lid. A total of 10 individuals were analyzed separately for the hydrostatic and atmospheric (control) pressure treatments. The pressure was gradually increased in steps of 50 bar at approximately 7-min intervals (Živaljić et al., 2020a) until the maximum pressure of 320 bar is reached. Thereafter, the pressure was directly released after 12 h, and the samples were examined under a Leica inverted microscope (Leica Instruments Inc.). The cells were considered dead when no movement was observed in both the body and cilia for at least 7 min (Živaljić et al., 2020a).

## Results

### ZooBank Registration

The present study and the newly described ciliate have been registered in the ZooBank database as follows:

Present study: urn:lsid:zoobank.org:pub:6CB50D24-E557-428A-B375-413218C45FBA.

Species: urn:lsid:zoobank.org:act:9BB86914-3CF9-43F0-86B6-8F18C275BA11.

### Morphological Description

Order Protocruziida Jankowski in Small and Lynn, 1985

Family Protocruziidae Jankowski in Small and Lynn, 1985

Genus *Protocruzia* Faria et al., 1922

**FIGURE 2:**
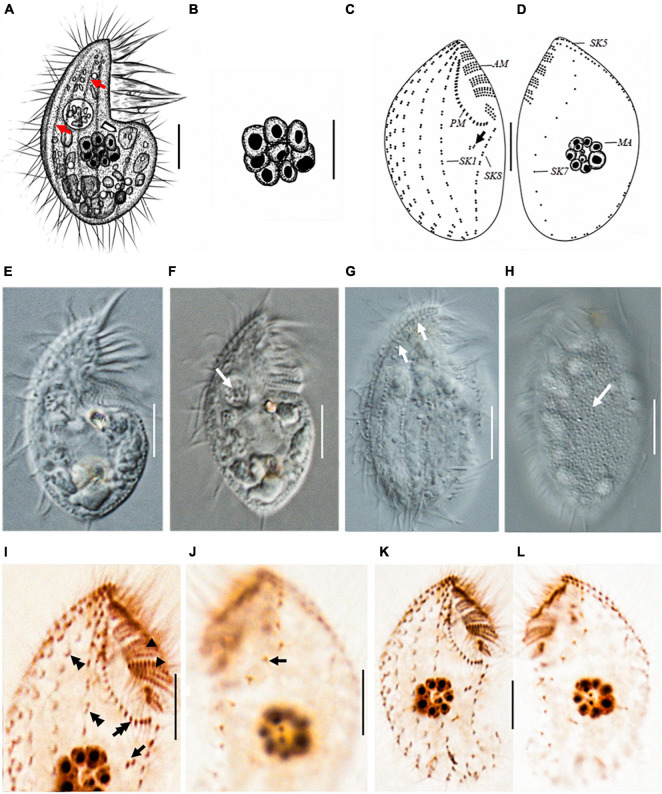
*Protocruzia marianaensis* sp. n. *in vivo* **(A,B,E–H)**, after protargol impregnation **(C,D,I–L)**. **(A,E–H)** Right lateral view of a representative individual. **(A,G)** Note the longitudinal furrows with the ciliary rows (arrows). **(B)** Macronucleus view. **(F)** Endoplasm of a squeezed cell; arrow points to a food vacuole. **(H)** Left lateral view shows the cortical granules (arrow). **(C,D,I–L)** Right **(C,I,K)** and left **(D,J,L)** lateral view of the holotype specimen, showing the ciliary pattern. Arrows in **(C,I)** mark postoral dikinetids. **(I)** Right anterior cell portion. Arrowheads point to adoral membranelles, the double arrow points to the paroral membrane, and the double arrowheads denote a dikinetid bearing two cilia. **(J)** Left anterior cell portion. The arrow denotes a monokinetid bearing two cilia. AM, adoral membranelles; MA, macronucleus; PM, paroral membrane; SK, somatic kinety; Scale bars = 5 μm.

*Protocruzia marianaensis* sp. n. (Figures 2A–L and Table 1)

**TABLE 1:** Morphometric data of *Protocruzia marianaensis* sp. n. based on *in vivo* and protargol-impregnated specimens.

**Characters**	**Min**	**Max**	**Mean**	**Median**	**SD**	**CV**	**n**
Body length (*in vivo*, μm)	25	32	28.8	28.7	2.1	7.2	20
Body width (*in vivo*, μm)	14	17	15.7	15.6	0.9	5.6	20
Buccal length (*in vivo*, μm)	10	12	10.7	10.6	0.7	6.1	20
Ratio of buccal length/body length (*in vivo*, %)	33	43	36.5	37.0	3.5	9.7	20
Body length (μm)	18	24	21.4	22.0	2.4	11.4	20
Body width (μm)	11	16	13.4	13.0	1.9	14.2	20
Buccal length (μm)	8	13	10.9	11.0	1.6	14.3	20
Ratio of buccal length/body length (%)	35	47	41.9	43.1	4.9	11.7	20
Number of adoral membranelles	6	6	6.0	6.0	0.0	0.0	20
Number of somatic kineties	8	8	8.0	8.0	0.0	0.0	20
Postoral dikinetids	1	3	2.0	2.0	0.7	42.5	20
Number of macronuclear globules	7	11	8.5	8.0	1.3	15.3	20
Macronuclei length (μm)	6	9	7.0	7.0	0.8	11.7	20
Macronuclei width (μm)	5	7	5.8	6.0	0.7	11.8	20

*CV, coefficient of variation in%; Max, maximum; Mean, arithmetic mean; Min, minimum; n, number of cells investigated; SD, standard deviation.*

#### Diagnosis

Marine *Protocruzia*, cell size *in vivo* approximately 25–32 × 14–17 μm; drop-shaped body in lateral view with a general length-to-width ratio of 2:1; the adoral zone occupies approximately 33–43% of cell length with six membranelles; eight somatic kineties; numerous colorless cortical granules densely scattered on the right side; and single macronucleus comprising 7–11 adjacent nuclear globules.

#### Type Locality

Deep-sea sediment samples from depths of 3,144 m (11°13′9″N, 139°55′50″E) and 3,092 m (11°47′49″N, 140°7′6″E), Mariana Trench area, Pacific Ocean.

#### Type Material

The protargol preparation containing the holotype (Figures 2K,L) has been deposited in the Laboratory of Protozoology, Institute of Evolution and Marine Biodiversity, Ocean University of China (registration number: LMJ2020121601-1).

#### Etymology

The specific name *marianaensis* refers to the Mariana Trench area type locality.

#### Gene Sequence

The 1,791-bp SSU rRNA gene sequence of *P. marianaensis* sp. n. has been deposited in GenBank under accession number MW114965.

### Morphological Characterization

Cell size *in vivo* approximately 25–32 × 14–17 μm; body length sometimes shortened to less than 16 μm during cultivation. Cells rigid with a drop-shaped outline in lateral view. Anterior end bluntly pointed, posterior end broadly rounded (Figure 2). Living cells scarcely contractile with a general length-to-width ratio of 2:1. Dorsal and ventral sides convex (Figures 2A,E,F). Longitudinal furrows along the ciliary rows on the right side (Figures 2A,G).

Numerous colorless or light gray cortical granules (∼0.2 μm across) densely scattered on the left side (Figure 2H). Cytoplasm hyaline, colorless, or slightly grayish. One food vacuole located near the buccal area (Figure 2F). No extrusomes detected *in vivo*. One macronucleus located at the center of the cell, composed of 7–11 adjacent globules (Figures 2A,B) and dispersed after protargol impregnation (Figures 2D,L). Micronucleus not visible *in vivo* or after protargol impregnation.

Buccal field concave, with oral cilia approximately 4–7 μm in length. Buccal apparatus extends to approximately half the body length (Figure 2E and Table 1). Invariably, six adoral membranelles bear approximately 180 cilia. Membranelles 1 (anterior-most) and 6 comprise two and three transverse rows of basal bodies, respectively, whereas other membranelles comprise four rows (Figure 2C). Paroral membrane commences anteriorly near the right side of membranelle 2, comprising two compact rows of basal bodies (Figures 2C,K).

Eight somatic kineties (Figure 2 and Table 1), six (kineties 1–5 and 8) on the right lateral side (Figures 2C,K), kinety 7 on the left lateral side, and kinety 6 along the edge (Figures 2D,L). Somatic cilia approximately 6 μm long, no elongated caudal cilium. Kineties 1–6 and 8 comprising closely arranged dikinetids (Figures 2C,I,K); kinety 7 consisting of sparse monokinetids (Figures 2J,L). Kineties 1–7 almost bipolar, and kinety 8 commencing at the posterior end of the paroral membrane, extending to the rear end of the cell (Figures 2C,D,K,L). Kinety 7 almost inversely S-shaped in the left lateral view (Figures 2D,L). One to three isolated dikinetids positioned post-orally near the anterior portion of kinety 8 (Figures 2C,I).

Locomotion usually involved crawling on substrate with occasional swimming, sometimes remaining momentarily stationary. When disturbed, cells moved rapidly toward upper waters, although in no fixed direction, rotating along the longitudinal axis or rapidly crawling anticlockwise (based on the right lateral view) on substrate. No cysts were observed.

### Sequence and Phylogenetic Position of *Protocruzia marianaensis* sp. n.

The sequence of the SSU rRNA gene, with length 1,791 bp and a GC content of 44.5%, has been deposited in the GenBank database under accession number MW114965. BLAST analysis revealed that the three sequences most closely similar to that of *P. marianaensis* sp. n. are all *Protocruzia* species, namely, those of *P. adhaerens* AY217727 (96.64% identity), *P. tuzeti* KU500620 (96.12% identity), and *P. contrax* DQ190467 (96.03% identity). Comparatively, sequence identities among these three species were higher than 98% (Supplementary Table 1).

**FIGURE 3:**
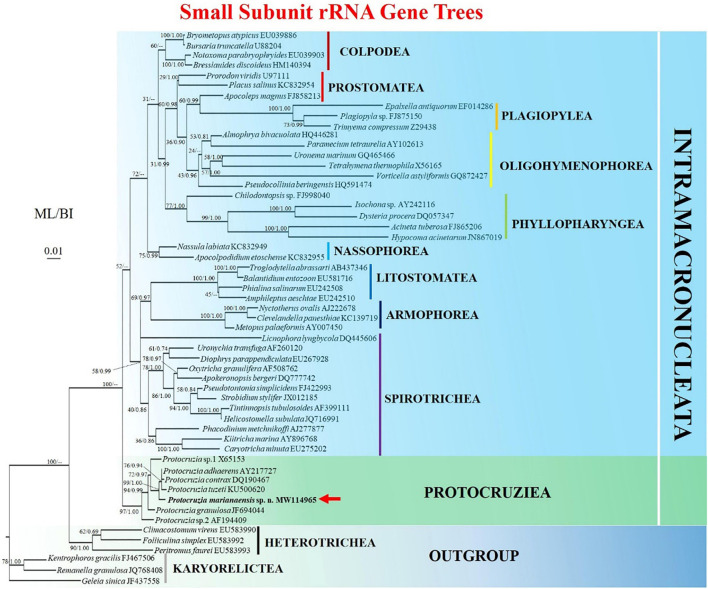
Maximum likelihood (ML) tree based on the small subunit ribosomal RNA gene showing the position of *Protocruzia marianaensis* sp. n. (in bold with red arrow). The GenBank accession numbers of the SSU rRNA sequences are given following the species names. Numerical support values are given at the respective nodes as maximum likelihood (ML) bootstrap percentages (1,000 replicates)/Bayesian posterior probabilities (BI). Clades with a different topology in the BI tree are indicated by “–.”Scale bar represents 0.01 expected substitutions.

The topologies of the ML and BI trees were found to be identical; thus, node support from both algorithms was mapped only onto the ML tree (Figure 3). All *Protocruzia* species were observed to cluster into an almost fully supported (97% ML, 1.00 BI) monophyletic clade at an early point of divergence between the subphyla Intramacronucleata and Postciliodesmatophora. *Protocruzia adhaerens* AY217727, *P. tuzeti* KU500620, and *P. contrax* DQ190467 were classified into a single clade (76% ML, 0.94 BI), to which *P. marianaensis* sp. n. was a sister taxon, and all four species clustered within a highly supported clade (99% ML, 1.00 BI).

### Survival at Deep-Sea Hydrostatic Pressures

To establish whether *P. marianaensis* sp. n. can withstand the hydrostatic pressures typically encountered in deep-sea areas, we conducted 10 independent survival experiments, with single specimens being exposed to increasing hydrostatic pressures. The maximum hydrostatic pressure applied (320 bar) is comparable to pressure conditions experienced at a depth of 3,200 m, which is approximately the depth at which samples were collected in the present study. At atmospheric pressure, cell locomotion was typically characterized by crawling on the substrate surface, punctuated by occasional spells of swimming. After 12 h under high-pressure conditions, all individuals were still alive, and we generally observed no substantial differences in their locomotory behavior. The only difference was the speed of locomotion in that the treated cells moved much slower than the cells in atmospheric pressure, especially when disturbance occurred (Supplementary Videos 1, 2). Moreover, we established that all examined *P. marianaensis* sp. n. individuals survived exposure to the maximum pressure of 320 bar without cyst formation.

## Discussion

### Comparison of *Protocruzia marianaensis* sp. n. With Congeners

Although it has been 100 years since Faria et al. (1922) initially established the genus *Protocruzia*, species in this genus have rarely been reported in previous studies, with only seven valid species being formally described, namely, *P. adhaerens* (Mansfeld, 1923) Kahl, 1932; *P. tuzeti* Villeneuve-Brachon, 1940; *P. contrax* (Mansfeld, 1923) Kahl, 1932; *P. depressa* Ammermann, 1968; *P. granulosa* Kahl, 1933; *P. labiata* Kahl, 1932; and *P. pigerrima* (Cohn, 1866) Faria et al., 1922. Moreover, only two of these species, namely, *P. tuzeti* and *P. granulosa*, have been sufficiently studied, with Jiang et al. (2016) presenting redescriptions of each.

**FIGURE 4:**
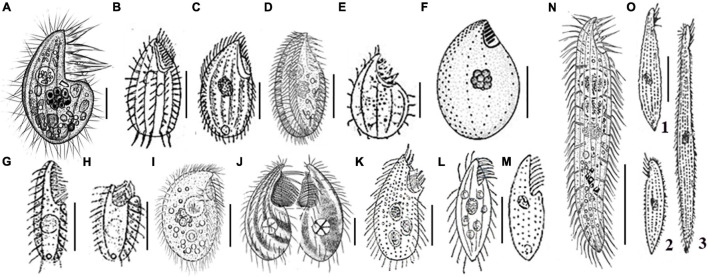
Eight *Protocruzia* species *in vivo* **(A–E,G–O)**, and after protargol impregnation **(F)**. **(A)** *Protocruzia marianaensis* sp. n. (from this study). **(B,C)** *Protocruzia adhaerens* (**B**, from Mansfeld, 1923; **C**, from Kahl, 1932). **(D–F)** *Protocruzia tuzeti* (**D**, from Jiang et al., 2016; **E**, from Villeneuve-Brachon, 1940; and **F**, from Grolière et al., 1980). **(G–I)** *Protocruzia contrax* (**G,H**, from Mansfeld, 1923; **I**, from Song and Wilbert, 1997). **(J)** *Protocruzia depressa* (from Ammermann, 1968). **(K)** *Protocruzia labiata* (from Kahl, 1932). **(L,M)** *Protocruzia pigerrima* (**L**, from Kahl, 1933; **M**, from Cohn, 1866). **(N,O)** *Protocruzia granulosa* (**N**, from Jiang et al., 2016; **O**, from Kahl, 1933), different cells from the same population (1: typical form; 2: non-extendable form; 3: extended form). Scale bars = 5 μm **(A)**, 10 μm **(E,G,J)**, 15 μm **(C,D,F,I,K–M)**, and 30 μm **(B,G,H,N,O)**.

In contrast with the aforementioned *Protocruzia* species, which have all been collected from different sea-surface habitats, the newly described *P. marianaensis* sp. n., which was collected from the Mariana Trench area at depths greater than 3,000 m, is the first *Protocruzia* species to be isolated from the deep. A comparison of the morphology of *P. marianaensis* sp. n. with that of several congeneric species revealed that, unlike the other *Protocruzia* species, the shape of *P. marianaensis* sp. n. cells is conspicuously more convex on the right side from the lateral view (Figure 4). Moreover, in *P. marianaensis* sp. n., the length of the buccal apparatus extends to almost half the body length (maximum 43%), which is notably higher than that observed in other *Protocruzia* species (Figure 4).

Based on the descriptions of *P. adhaerens* presented by Mansfeld (1923) and Kahl (1932), it would appear that this species is most closely related to *P. marianaensis* sp. n., although there are several clear interspecific differences, including (i) the larger body size of *P. adhaerens* (∼63 × 34 μm from Mansfeld, 1923 and ∼41 × 22 μm from Kahl, 1933 vs. 25–32 × 14–17 μm in *P. marianaensis* sp. n.); (ii) more adoral membranelles (9–10 vs. 6 in *P. marianaensis* sp. n.); (iii) fewer somatic kineties (5 or 6 vs. invariant 8 in *P. marianaensis* sp. n.).

Among the other verified *Protocruzia* species, Villeneuve-Brachon (1940) presented the first, albeit succinct, description of *P. tuzeti*, with Grolière et al. (1980) and Jiang et al. (2016) subsequently redescribing their morphology in greater detail based on specimens collected in the Black Sea and the Shenzhen region of China, respectively. The Black Sea population (Figure 4F) is clearly distinct from *P. marianaensis* sp. n. with respect to body shape, larger cell size (30–35 × 17–19 vs. 25–32 × 14–17 μm), and a larger number of somatic kineties (10–11 vs. 8). Generally, however, *P. marianaensis* sp. n. is more similar to the *P. tuzeti* from Shenzhen (Figure 4D), in that both species are characterized by the presence of six adoral membranelles and eight somatic kineties. However, differences in other characters clearly establish the two isolates as different species. For example, compared with *P. marianaensis* sp. n., *P. tuzeti* has (i) a larger cell size (30–40 × 12–25 vs. 25–32 × 14–17 μm); (ii) different patterns of somatic kinety 7 (dikinetids in *P. tuzeti* vs. mostly monokinetids in *P. marianaensis* sp. n.); and (iii) a larger number of nuclear globules (13–19 in *P. tuzeti* vs. 7–11 in *P. marianaensis* sp. n.).

*Protocruzia contrax* (Mansfeld, 1923) Kahl, 1932, was originally described as highly contractile, with 11 membranelles and two kineties based on the observation of live cells (Mansfeld, 1923; Kahl, 1932). In addition, Song and Wilbert (1997) described a species sampled from Qingdao with 9–12 somatic kineties as *P. contrax*; however, Jiang et al. (2016) subsequently reasoned that this study had misidentified the species. Nevertheless, *P. marianaensis* sp. n. can still be distinguished from the *P. contrax* isolates described by Song and Wilbert (1997); Mansfeld (1923), and Kahl (1932) by the presence of cortical granules and the number of somatic kineties.

Ammermann (1968) described *P. depressa* from the North Sea, which is again distinct from *P. marianaensis* sp. n. with respect to (i) a larger cell size (40–50 × 15–30 vs. 25–32 × 14–17 μm) and (ii) fewer kineties (5 vs. 8). Moreover, *P. depressa* has a conspicuously pointed anterior end (Figure 4J).

*Protocruzia granulosa* was first discovered by Kahl (1933) in the North Sea off Heligoland and was subsequently redescribed by Jiang et al. (2016). Both descriptions clearly indicate that there are significant differences between *P. granulosa* and *P. marianaensis* sp. n. (Figures 4A,N,O), with the former being characterized by (i) a longer cell length (60–180 vs. 25–32 μm); (ii) a highly contractile body (vs. rarely contractile in *P. marianaensis* sp. n.); (iii) a larger number of somatic kineties (13–14 vs. 8); (iv) an absence of dikinetids (vs. 1–3); and (v) a larger number of nuclear globules (17–28 vs. 7–11).

*Protocruzia labiata* Kahl, 1932, has been cursorily reported from Germany without any description of the ciliary pattern. Kahl (1932) emphasized that the adoral membranelles of this species form two groups (Figure 4K), which was different from *P. marianaensis* sp. n. However, *P. labiata* should be reinvestigated to confirm its validity.

*Protocruzia pigerrima* (Cohn, 1866) Faria, Cunha, and Pinto has been briefly described as having a “flat body with thin and long cilia arranged in longitudinal lines; groove-shaped peristoma starting from the anterior end to about the middle of the body, presenting on its edge a series of cilia longer and thicker than those in the lining of the body” (Faria et al., 1922). Although the ciliary pattern of this species is unclear, drawings indicate a pointed rear end and a distinct flat body, which differentiates it from *P. marianaensis* sp. n. characterized by a rounded rear end (Figures 4L,M).

### Phylogenetic Position of *Protocruzia marianaensis* sp. n.

Over the past few decades, *Protocruzia* species have variously been placed in either the class Karyorelictea or Heterotrichea (Ammermann, 1968; Ruthmann and Hauser, 1974; Small and Lynn, 1981; de Puytorac, 1994; Foissner, 1996; Song and Wilbert, 1997). However, the findings of recent studies have provided sufficient evidence to indicate that these species should be classified in an independent class (Li et al., 2010; Gentekaki et al., 2014; Gao et al., 2016; Jiang et al., 2016; Sheng et al., 2018). Consistently, the results of phylogenetic analyses performed in the present study would tend to indicate the uniqueness of *Protocruzia* species. The phylogenetic trees obtained based on both ML and BI analyses of the SSU rRNA dataset clearly indicated that the species of *Protocruzia* cluster into a nearly fully supported (97% ML, 1.00 BI) monophyletic group. Accordingly, given that these species form an independent clade distinct from that of other groups and taking into consideration the aforementioned differences in morphological traits, we tend to concur with previous suggestions that *Protocruzia* should be regarded as a separate lineage at the class level. Moreover, we established that *P. marianaensis* sp. n. mapped along a distinct branch most closely allied to that containing *P. adhaerens*, *P. tuzeti*, and *P. contrax*. At the molecular level, however, the identity between *P. marianaensis* sp. n. and these most closely related species is at its highest only at 96.64%. This compares with the lowest identity of 98.17% among *P. adhaerens*, *P. tuzeti*, and *P. contrax* (Supplementary Table 1), which are generally validated as discrete species. We accordingly believe that the molecular data provide sufficiently convincing evidence to indicate that *P. marianaensis* sp. n. is a new species within the genus *Protocruzia*.

### Survival Analysis of *Protocruzia marianaensis* sp. n. at Hydrostatic Pressures

Although there is relatively little data on ciliates isolated from deep-sea areas (Schoenle et al., 2017; Živaljić et al., 2020a,b), the findings of several studies have indicated that ciliates may form a remarkably diverse component of deep-sea communities. Schoenle et al. (2017), for example, have shown that deep-sea strains of *Pseudocohnilembus persalinus* and *Uronema* sp. can survive at 557 bar, whereas Živaljić et al. (2020b) established that the former of these two species could only survive at pressures up to 300 bar. Similarly, the deep-sea ciliates *Euplotes dominicanus* and *Aristerostoma* sp. have been found to maintain activity at high hydrostatic pressures of up to 500 and 400 bar, respectively (Živaljić et al., 2020b). The *P. marianaensis* sp. n. isolated in the present study is, to the best of our knowledge, the first *Protocruzia* species to be collected from the deep sea, and we established that specimens of this isolate survive exposure to both atmospheric pressure and high hydrostatic pressure up to 320 bar. Interestingly, however, we found no evidence to indicate cyst formation either in the hydrostatic pressure experiment or in cultures maintained at atmospheric pressure. These observations are consistent with those reported for *E. dominicanus* (Živaljić et al., 2020a,b), although in contrast with those of *P. persalinus* and *Aristerostoma* sp., which were observed to undergo cyst formation when exposed to high hydrostatic pressure (Schoenle et al., 2017; Živaljić et al., 2020b). These findings would thus tend to indicate that *P. marianaensis* sp. n. maintain activity at the depths at which specimens were originally collected. In the present study, it should be noted that the hydrostatic pressure caused a slowdown in the reactions of *Protocruzia* cells, and this may be a strategy for these organisms to recover from a high-pressure environment. Clearly, the most reliable approach to establishing the actual status of ciliates inhabiting deep-sea regions is to microscopically examine *in situ* sediment samples under pressure. Additionally, it would be of considerable interest to determine the mechanisms underlying the wide adaptability of these organisms to a high-pressure environment.

## Data Availability Statement

The datasets presented in this study can be found in online repositories. The names of the repository/repositories and accession number(s) can be found in the article/Supplementary Material.

## Author Contributions

CL was involved in the cultivation of ciliates, performed live observations, protargol staining, pressure experiments, and wrote the manuscript. WW performed the PCR analysis and molecular cloning and contributed to the revision of the manuscript. LD participated in sediment sampling and contributed to the revision of the manuscript. IM contributed to the revision of the manuscript and improved the style, and english grammar. JC and FW supervised the study and contributed to the revision of the manuscript. All authors contributed to the article and approved the submitted version.

## Conflict of Interest

The authors declare that the research was conducted in the absence of any commercial or financial relationships that could be construed as a potential conflict of interest.

## Publisher’s Note

All claims expressed in this article are solely those of the authors and do not necessarily represent those of their affiliated organizations, or those of the publisher, the editors and the reviewers. Any product that may be evaluated in this article, or claim that may be made by its manufacturer, is not guaranteed or endorsed by the publisher.

## Supplementary Material

Supplementary Figure 1 Hydrostatic pressure system. **(A)** Arrow points to the pressure control console, and double arrow points to the stainless-steel chamber. **(B)** Air compressor. **(C)** Eppendorf tubes with sample.

Supplementary Table 1 Sequence identities among four *Protocruzia* species.

Supplementary Video 1 *Protocruzia* cells in atmospheric pressure.

Supplementary Video 2 *Protocruzia* cells after high-pressure treatment.
