# Supplementary material for: A New Protocruzia Species (Ciliophora: Protocruziida) Isolated From the Mariana Trench Area
Source: Front Microbiol. 2021 Oct 20;12:743920. doi: 10.3389/fmicb.2021.743920 (PMC8564289; doi:10.3389/fmicb.2021.743920)
Supplement: Supplementary Figure 1 — Hydrostatic pressure system. (A) Arrow points to the pressure control console, and double arrow points to the stainless-steel chamber. (B) Air compressor. (C) Eppendorf tubes with sample. [file Data_Sheet_1.docx]

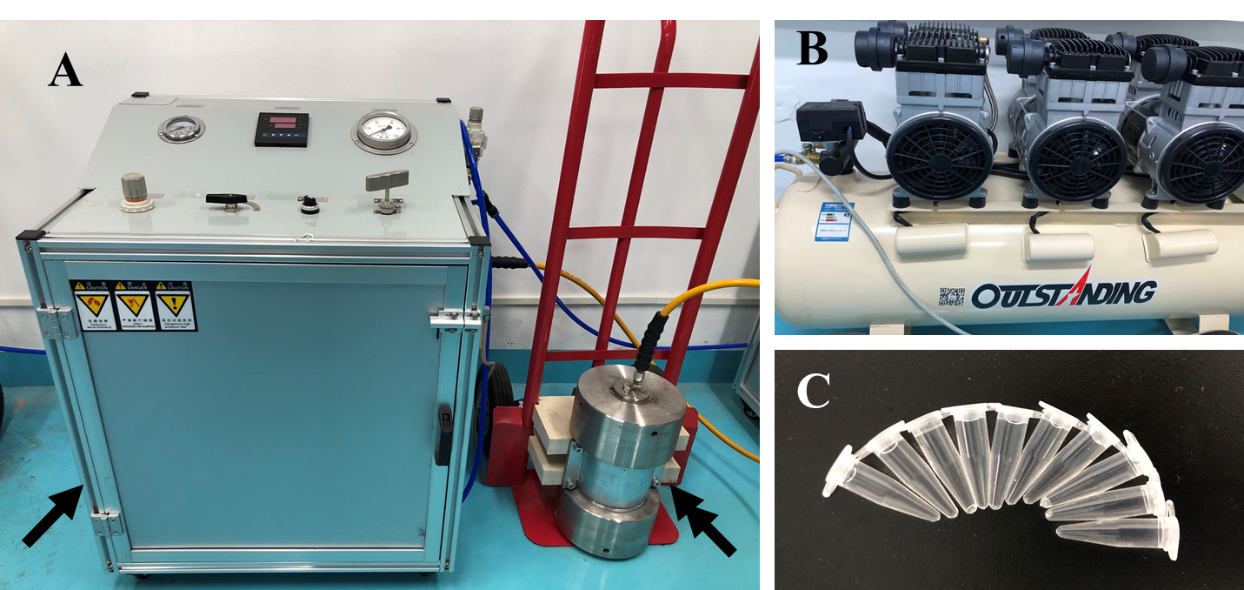


**FIGURE 1S** Hydrostatic pressure system. (A) Arrow points to the pressure control console and double-arrow points to the stainless-steel chamber. (B) Air compressor. (C) Eppendorf tubes with sample.

**Table S1** Sequence identities among four *Protocruzia* species

| Species | *Protocruzia marianaensis* sp. n.  MW114965 | *P. adhaerens*  AY217727 | *P. tuzeti*  KU500620 | *P. contrax*  DQ190467 |
| --- | --- | --- | --- | --- |
| *Protocruzia marianaensis* sp. n.  MW114965 | 100% | 96.64% | 96.12% | 96.03% |
| *P. adhaerens* AY217727 | 96.64% | 100% | 98.17% | 98.64% |
| *P. tuzeti* KU500620 | 96.12% | 98.17% | 100% | 98.99% |
| *P. contrax* DQ190467 | 96.03% | 98.64% | 98.99% | 100% |
